# Urine as matrix for analysis of neurofilament light chain is not suitable to distinguish frontotemporal dementia from psychiatric diseases

**DOI:** 10.1093/braincomms/fcad120

**Published:** 2023-04-12

**Authors:** Marie-Paule E van Engelen, Hans Heijst, Eline A J Willemse, Mardien L Oudega, Lisa Vermunt, Philip Scheltens, Everard G B Vijverberg, Yolande A L Pijnenburg, Charlotte E Teunissen

**Affiliations:** Alzheimer Center Amsterdam, Neurology, Vrije Universiteit Amsterdam, Amsterdam UMC location VUmc, Amsterdam, Noord-Holland 1081 HZ, The Netherlands; Amsterdam Neuroscience, Neurodegeneration, Amsterdam, Noord-Holland 1081 HV, The Netherlands; Neurochemistry Laboratory, Department of Clinical Chemistry, Amsterdam Neuroscience, Vrije Universiteit Amsterdam, Amsterdam UMC, 1081 HV, Amsterdam, The Netherlands; Neurochemistry Laboratory, Department of Clinical Chemistry, Amsterdam Neuroscience, Vrije Universiteit Amsterdam, Amsterdam UMC, 1081 HV, Amsterdam, The Netherlands; Department of Neurology, University Hospital Basel and University of Basel, 4031, Basel, Switzerland; Alzheimer Center Amsterdam, Neurology, Vrije Universiteit Amsterdam, Amsterdam UMC location VUmc, Amsterdam, Noord-Holland 1081 HZ, The Netherlands; Amsterdam Neuroscience, Neurodegeneration, Amsterdam, Noord-Holland 1081 HV, The Netherlands; GGZ inGeest Specialized Mental Health Care, Location De Nieuwe Valerius, Amsterdam, Noord-Holland 1070 BB, The Netherlands; Department of Psychiatry, Amsterdam UMC location Vrije Universiteit Amsterdam, Amsterdam, Noord-Holland 1081 HV, The Netherlands; Amsterdam Neuroscience, Mood Anxiety Psychosis Sleep & Stress program, Amsterdam, Noord-Holland 1081 HV, The Netherlands; Amsterdam Neuroscience, Neurodegeneration, Amsterdam, Noord-Holland 1081 HV, The Netherlands; Neurochemistry Laboratory, Department of Clinical Chemistry, Amsterdam Neuroscience, Vrije Universiteit Amsterdam, Amsterdam UMC, 1081 HV, Amsterdam, The Netherlands; Alzheimer Center Amsterdam, Neurology, Vrije Universiteit Amsterdam, Amsterdam UMC location VUmc, Amsterdam, Noord-Holland 1081 HZ, The Netherlands; Amsterdam Neuroscience, Neurodegeneration, Amsterdam, Noord-Holland 1081 HV, The Netherlands; Alzheimer Center Amsterdam, Neurology, Vrije Universiteit Amsterdam, Amsterdam UMC location VUmc, Amsterdam, Noord-Holland 1081 HZ, The Netherlands; Amsterdam Neuroscience, Neurodegeneration, Amsterdam, Noord-Holland 1081 HV, The Netherlands; Alzheimer Center Amsterdam, Neurology, Vrije Universiteit Amsterdam, Amsterdam UMC location VUmc, Amsterdam, Noord-Holland 1081 HZ, The Netherlands; Amsterdam Neuroscience, Neurodegeneration, Amsterdam, Noord-Holland 1081 HV, The Netherlands; Amsterdam Neuroscience, Neurodegeneration, Amsterdam, Noord-Holland 1081 HV, The Netherlands; Neurochemistry Laboratory, Department of Clinical Chemistry, Amsterdam Neuroscience, Vrije Universiteit Amsterdam, Amsterdam UMC, 1081 HV, Amsterdam, The Netherlands

**Keywords:** neurofilament light chain, urine, diagnostics, frontotemporal dementia, psychiatric disorders

## Abstract

**The** clinical overlap of frontotemporal dementia and primary psychiatric diseases hampers diagnostic distinction, leading to frequent misdiagnosis and diagnostic delay. Neurofilament light chain has shown great potential in CSF and blood for the distinction of frontotemporal dementia from primary psychiatric diseases. Measurement of neurofilament light chain in urine would be even more patient-friendly. We aimed to test the performance of neurofilament light chain urine measurements for diagnostics in frontotemporal dementia and to assess their correlation with serum levels. Fifty-five subjects (*n* = 19 frontotemporal dementia, *n* = 19 primary psychiatric diseases and *n* = 17 controls) were included with available paired urine and serum samples. All subjects underwent standardized extensive diagnostic assessment. Samples were analysed with the ultrasensitive single molecule array neurofilament light chain assay. Neurofilament light chain group comparisons were performed adjusted for age, sex and geriatric depression scale. In the majority of the cohort, neurofilament light chain concentrations were not detectable in urine (*n* = 6 samples above lower limit of detection (0.038 pg/ml): *n* = 5 frontotemporal dementia, *n* = 1 primary psychiatric disease). The frequency of a detectable neurofilament light chain level in urine in the frontotemporal dementia group did not differ from psychiatric disorders (Fisher Exact-test *P* = 0.180). In the individuals with detectable urine neurofilament light chain values, there was no correlation between the urine and serum neurofilament light chain levels. As expected, serum neurofilament light chain levels were higher in frontotemporal dementia compared to primary psychiatric diseases and controls (*P* < 0.001), adjusted for age, sex and geriatric depression scale. Receiver operating characteristic curve analysis of serum neurofilament light chain of frontotemporal dementia versus primary psychiatric diseases showed an area under the curve of 0.978 95% confidence interval 0.941–1.000, *P* < 0.001. Urine is not suitable as a matrix for neurofilament light chain analysis and serum neurofilament light chain is still the most patient-friendly matrix for differentiation between frontotemporal dementia and primary psychiatric diseases.

## Teaser

Van Engelen *et al*. report that the use of urine as a matrix for neurofilament light chain analysis to differentiate frontotemporal dementia from psychiatric disorders is not suitable and that urine neurofilament light chain levels did not correlate with their levels in serum.

## Graphical Abstract

**Graphical abstract:**
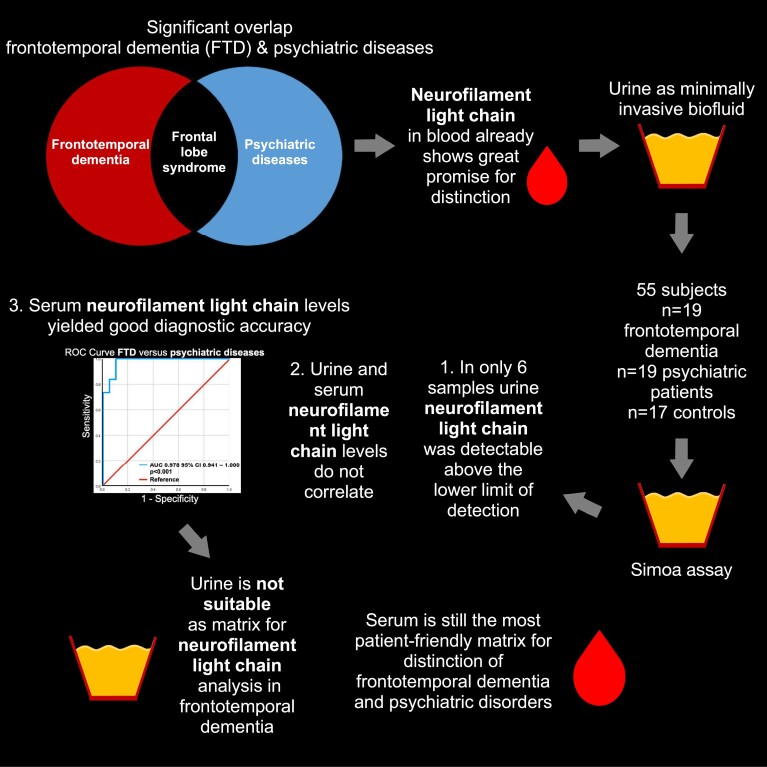

## Introduction

Due to the significant overlap of clinical symptoms and overlapping frontotemporal hypometabolic patterns on functional neuroimaging, the diagnostic distinction between frontotemporal dementia (FTD) and primary psychiatric diseases (PPD) is challenging, leading to misdiagnosis in 50% of the cases and an approximate delay of 6.4 years till correct diagnosis.^1^

Contrary to the well-validated cerebrospinal fluid (CSF) biomarkers in Alzheimer’s disease, an early specific fluid biomarker for FTD is lacking.^2^ Emerging studies report elevated levels of neurofilament light chain (NfL) in FTD than in various other neurodegenerative disorders, indicating a role as a diagnostic biomarker. Moreover, multiple studies already showed that NfL has the potential to discriminate FTD from PPD.^3–5^

To our knowledge, there are no previous studies that used urine as a matrix for NfL analysis. Therefore, we first performed a method validation for the detection of NfL in urine, using the urine of cancer patients, in which NfL was detectable in low concentrations (unpublished results). Assessment of urine NfL would be a minimal invasive, more patient-friendly and feasible procedure, compared to a lumbar puncture to collect CSF or a vena-puncture to collect blood. The latter two procedures can lead to complications and require a trained, authorized and competent care professional, contrary to the self-collection of urine. In addition, a minimally invasive biomarker could contribute to distinguishing FTD from PPD in early disease stages, enabling the start of (future) therapies in an early phase.

Therefore, the aim of this study was to test the feasibility of NfL measurements in urine for diagnostics in FTD and to assess if urine and serum NfL levels correlate.

## Methods

### Participants

Patients with FTD, PPD and controls were included with available paired urine and blood serum collection of the same date, between 45 and 75 years of age and the groups were matched for age and sex. Participants were part of the Alzheimer Dementia Cohort^6^ and were recruited during their diagnostic visit at the memory clinic of the Alzheimer Center of Amsterdam between February 2010 and January 2012. Urine and serum samples were collected during the first diagnostic visit at baseline.

The study was approved by the Medical Ethical Committee of Amsterdam University Medical Center (UMC) and all participants provided written and oral informed consent. The study has been carried out in accordance with the Declaration of Helsinki.

### Diagnostic procedure

All participants underwent a standardized diagnostic procedure at baseline including a clinical assessment by a cognitive neurologist, behavioural [frontal assessment battery (FAB)] cognitive [mini mental state examination (MMSE)] and mood [geriatric depression scale (GDS)] screening tests, neuropsychological tests assessing all cognitive domains (attention, concentration, speed, memory, executive functioning, language and visual spatial functioning), blood examination to rule out somatic causes, biomarker assessment in CSF, EEG and neuroimaging: MRI, and if indicated, a [^18^F]FDG-PET scan.^6^ Diagnosis was established in a multidisciplinary meeting using consensus criteria for FTD^7^ and PPD diagnostic and statistical manual of mental disorders (DSM)-V.^8^ If a psychiatric disease was suspected, the patient was referred to a psychiatrist for further psychiatric assessment. The median (clinical) follow-up duration for all patients was 16[4–38] months during which their diagnosis was confirmed (Table 1).

**Table 1:** Demographics

	All	FTD	PPD	Controls	*P-*value
	*n* = 55	*n* = 19	*n* = 19	*n* = 17	
Sex (female, %)	17(31%)	6(11%)	7(13%)	4(7%)	0.713^b^
Age (mean ± SD)	60 ± 6.4	62 ± 7.1	58 ± 4.5	60 ± 6.6	0.048^c^
Follow-up duration, months; median[Q1–Q3]	16[4–38]	16[3–62]	5[0–33]	25[12–44.5]	0.093^e^
Disease duration, years; median[Q1–Q3]^a^	2[2–4]	2.5[2–6]	2[2–4]	n.a.	0.727^d^
GDS median[Q1–Q3]	3[2–6]	4[2–5]	8[3–11]	2[1–3]	0.002^e^
MMSE median[Q1–Q3]	28[24–29]	24[21–29]	28[25–29]	29[28–30]	0.005^e^
FAB median[Q1–Q3]	17[14.5–18]	15[13–17]	17[14–18]	17[16–18]	0.070^e^

a Represents the time from onset of symptoms till performance of NfL Simoa assay in blood serum and urine.

b Fisher exact-test.

c Analysis of variance (ANOVA).

d Mann–Whitney U Test.

e Kruskal–Wallis Test.

All FTD patients (*n* = 19) fulfilled diagnostic criteria for probable (*n* = 9) or definite FTD (*n* = 1),^7^ semantic dementia (*n* = 5), or right temporal variant of FTD^9^ (*n* = 4). PPD were diagnosed with a functional neurologic disorder *n* = 2; obsessive-compulsive disorder *n* = 1; unspecified mental disorder *n* = 1; bipolar disorder, depressive phase *n* = 1; depression, *n* = 6; adjustment disorder *n* = 3; anxiety disorder *n* = 1; autism spectrum disorder *n* = 1; personality disorder *n* = 2; somatic symptom disorder *n* = 2.^8^

Abbreviations: n.a. = not applicable, MMSE = mini mental state exam, FAB = frontal assessment battery, GDS = geriatric depression scale, PPD = primary psychiatric disorders.

All FTD patients fulfilled the diagnostic criteria for probable (*n* = 9) or definite behavioural variant of FTD^7^ (*n* = 1, diagnosis on base of brain autopsy), semantic dementia (*n* = 5) or right temporal variant of FTD^9^ (*n* = 4).

All PPD had a active psychiatric disease at the time of the urine assessment. PPD was diagnosed with a functional neurologic disorder *n* = 2; obsessive-compulsive disorder *n* = 1; unspecified mental disorder *n* = 1; bipolar disorder, depressive phase *n* = 1; depression, *n* = 6; adjustment disorder *n* = 3; anxiety disorder *n* = 1; autism spectrum disorder *n* = 1; personality disorder *n* = 2; somatic symptom disorder *n* = 2.^8^ As a control group, participants with subjective cognitive decline^10^ (*n* = 17) were included that had no current or recent psychiatric nor a neurodegenerative disorder based on previously described standardized diagnostic assessment.^6^ Excluded were participants with acute (recent) myocardial infarction, cerebral ischaemic events or neuroinflammation, active peripheral neurological diseases, urological disease, kidney disease, or a recent intensive care admission, evaluated from the patient records, as these factors might influence (urine) NfL concentration.^11,12^ The CSF of all patients was negative for Alzheimer’s disease biomarkers decreased amyloid β 42 and increased tau and phosphorylated tau levels.^13^

### Urine collection

Urine samples were collected at baseline during the visit to the memory clinic in polystyrene containers (Avantor) and centrifuged for 10 minutes at 1800*g* at 4°C and stored in cryovials (Sarstedt, 1 ml) at −80°C in the Amsterdam UMC Biobank. The effect of freezing/thawing cycli for Nfl in urine is unknown, therefore we used fresh material that had not undergone freeze/thaw cycles before our analysis. *Off note*: urine was not collected in the morning.

### Simoa NfL assay

Analysis of NfL concentration was performed with the NfL single molecule array (Simoa) kit (Quanterix^®^, Billerica, USA) according to the manufacturer’s instructions. In brief, all samples were spun at 10 000*×g* for 5 minutes to precipitate debris and transferred to wells in 96-well Quanterix^®^ plates for duplicate tests and to run with on-board 4 × dilution (serum) and 2 × dilution (urine). Analysis in urine was performed in FTD and PPD (*n* = 38) to assess if NfL was detectable in urine and inter-assay variation was monitored using internal quality controls (serum) at two levels.

### Statistical analysis

Statistical analyses were performed in IBM SPSS Statistics for Windows Version 26.0. Choice of test (ANOVA), Kruskal–Wallis test was depending on the distribution of the continuous variables and categorical variables were analysed with the Fisher’s Exact-test. Multivariable regression analysis was performed to assess the relation between NfL concentration and diagnosis, adjusted for age, sex and GDS. Spearman correlation coefficients were calculated to test for the correlation of NfL concentration between serum and urine. Discrimination accuracies of NfL between FTD, PPD and controls were determined with ROC analysis.

## Results

### Demographics

FTD patients were older than PPD (FTD 62 ± 7.1; PPD 58 ± 4.5; controls 60 ± 6.6; *P* = 0.048) and FTD patients had lower mini mental state exam (MMSE) scores compared to controls [FTD 28(24–29); PPD 28(25–29); controls 29(28–30); *P* = 0.005]. PPD had higher scores on the GDS compared to FTD and controls [FTD 4(2–5); PPD 8(3–11); controls 2(1–3); *P* = 0.002]. There were no group differences in sex [females, FTD 6 (11%); PPD 7 (13%); controls 4(7%), *P* = 0.713], disease duration in years [FTD 2(2–6); PPD 2(2–4); *P* = 0.727], follow-up duration in months [FTD 16(3–62); PPD 5(0–33); controls 25(12–44.5); *P* = 0.093] and FAB total scores [FTD 15(13–17); PPD 17(14–18); controls 17(16–18); *P* = 0.070] (Table 1).

### Urine NfL

**Figure 1:**
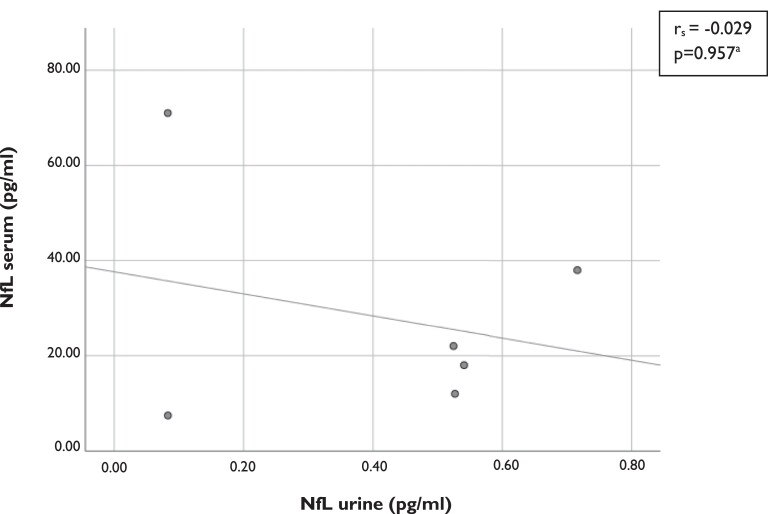
**Caption/legend: correlation paired urine and serum NfL.** Data represents NfL (pg/ml) in serum (*y*-axis) and urine (*x*-axis) of each patient (sphere) in which urine NfL was detected, in the cohort of FTD and PPD patients (*n* = 38). ^a^Spearman correlation. Abbreviations: NfL = neurofilament light chain; SD = semantic dementia; RtvFTD = right temporal variant of frontotemporal dementia.

NfL was detectable in 6 urine samples (*n* = 5 FTD; *n* = 1 PPD), whereas in the other samples, the NfL concentration was below the lower limit of detection (LOD): 0.038 pg/ml. Therefore, further analysis of NfL in urine was not performed in controls. Spearman correlation analysis of paired urine and serum did not yield significant results (*P* = 0.957, Fig. 1). The distribution of diagnosis in cases in which urine NfL was detected (*n* = 5 FTD, *n* = 1 PPD) was not different from random (*P* = 0.180). Table 2 represents the raw data of paired urine and serum samples.

**Table 2:** Raw data of paired urine and serum samples

Raw data of paired urine and serum samples			Ranked serum NfL levels with paired urine NfL		
Subjects, diagnosis	Urine NfL pg/ml	Serum NfL pg/ml	Subjects, diagnosis	*n* = 10 highest serum NfL pg/ml, ranked	Urine NfL pg/ml
Psychiatry, functional disorder	0.08	7.46	Probable bvFTD	71.00	0.08
Probable bvFTD	0.53	12.00	SD	63.00	–
Probable bvFTD	0.52	22.00	SD	56.00	–
RtvFTD	0.08	71.00	SD	46.00	–
RtvFTD	0.72	38.00	RtvFTD	38.00	0.72
Definite bvFTD	0.54	18.00		37.00	–
			Probable bvFTD	36.00	_
			SD	25.00	–
			Probable bvFTD	22.00	0.52
			RtvFTD	21.00	–

Abbreviations: bvFTD = behavioural variant of frontotemporal dementia; NfL = neurofilament light chain; SD = semantic dementia; RtvFTD = right temporal variant of frontotemporal dementia. Data represents (I) raw data of paired urine and serum NfL (pg/ml) and (II) ranked serum NfL levels with paired urine NfL.

### Serum NfL

**Figure 2:**
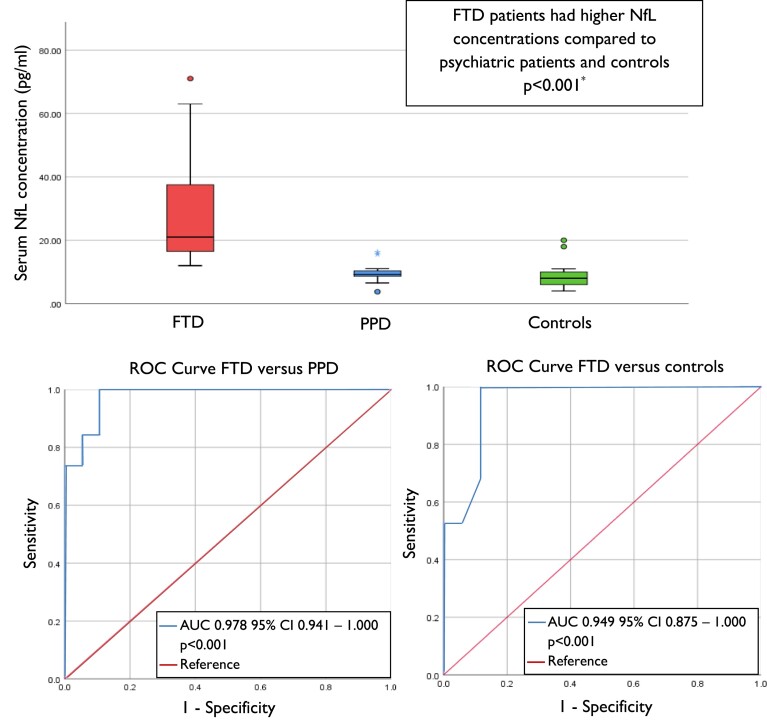
**Caption/legend: group differences in serum NfL concentration and diagnostic accuracy.** Data represents group differences (*n* = 55) in serum NfL (*Kruskal–Wallis Test) and its diagnostic accuracy (ROC analysis). Outliers are indicated as small circles (outlier: 3rd quartile + 1.5*interquartile range or 1st quartile—1.5*interquartile range) or asterisk (extreme outlier: 3rd quartile + 3*interquartile range or 1st quartile—3*interquartile range). Abbreviations: FTD frontotemporal dementia, PPD = primary psychiatric diseases. NfL = neurofilament light chain, ROC = receiver operating curve.

FTD patients had a higher mean serum NfL concentration compared to PPD and controls (FTD 29.3 ± 18.0 pg/ml versus PPD 9.5 ± 2.8 pg/ml versus controls 8.9 ± 4.3 pg/ml, *P* < 0.001, adjusted for age, sex and GDS, explained variance 61%, Fig. 2). There was also a significant positive relation between age and serum NfL concentration (log B coefficient: 0.026, *P* = 0.009).

### Diagnostic accuracy serum NfL

ROC analysis of serum NfL in FTD versus controls showed an area under the curve (AUC) of 0.95 (95% CI 0.88–1.00 *P* < 0.001, Fig. 2). Comparing serum NfL concentration of FTD to PPD, ROC analysis showed an AUC of 0.978, 95% CI 0.941–1.000 *P* < 0.001, with an optimal cut off value of 11.5 pg/ml (sensitivity 100%, specificity 90%).

## Discussion

Our results show that urine is not suitable as a matrix for NfL analysis for diagnostics in FTD and that urine and serum NfL levels did not correlate. Serum NfL provided an accurate distinction between FTD and PPD. These results thus indicate that despite using the most sensitive technology, measurement of NfL in urine cannot replace serum. Also, preliminary data in our lab showed that tear fluid is unsuitable for NfL analysis (unpublished results). Therefore, blood (serum/plasma) NfL is still the most patient-friendly matrix for differentiation, as reported before.^4,5^

Reasons for urine NfL not reaching the LOD might be that NfL does not reach sufficiently measurable levels in urine. Total urinary protein excretion in healthy adults should be less than 150 mg/day and higher rates of protein excretion often reflect a pathological increase in glomerular permeability that allows the filtration of macromolecules such as albumin.^14^ In this cohort, participants with urological or kidney diseases were excluded (*off note*: data on renal function was not available). As NfL is also a macromolecule and assuming that it appears in urine intact, it is conceivable that in patients with an adequate urinary tract system such as in this cohort, NfL gets largely filtered by the glomerular capillary wall thereby preventing urine NfL excretion, even in patients with high NfL levels. Also, we did not collect morning urine which is more concentrated compassing of probably higher NfL concentration, compared to urine produced during the day.

This FTD cohort with right temporal variant FTD and semantic dementia patients had a high probability of underlying TAR DNA-binding protein 43 (TDP-43) pathology,^15^ which is associated with increased NfL levels.^16^ As in FTD patients the NfL concentration is significantly higher compared to other neurodegenerative diseases, in particular those FTD patients with underlying TDP-43 pathology, this FTD cohort has the highest likelihood for detection of NfL in urine. It is thus inconceivable that urine NfL is detectable in other diagnostic FTD groups. Notwithstanding, the sensitivity of the measuring platform is also a determining factor for detection. Recently, reliable quantification of NfL became possible with the introduction of the Simoa assays in blood and CSF, across the full range of concentrations including in healthy participants.^17^ Our results suggest that an even more sensitive future technology might be needed to detect NfL in urine since we did find an NfL signal in a few FTD patients.

Since urine is unsuitable as a matrix for NfL analysis, serum NfL is the current matrix of choice for the implementation of NfL analysis to distinguish FTD from PPD or controls in clinical practice.

## Data Availability

The data and materials that support the findings of this study are available from the corresponding author on reasonable request.

## Abbreviations

AUC =: area under the curve
CI =: confidence interval
CSF =: cerebrospinal fluid
DSM =: Diagnostic and Statistical Manual of Mental Disorders
FAB =: frontal assessment battery
FTD =: frontotemporal dementia
GDS =: geriatric depression scale
LOD =: lower limit of detection
MMSE =: Mini Mental State Exam
NfL =: neurofilament light chain
PPD =: primary psychiatric diseases
PRODIA =: Proteomics for differential diagnosis of Alzheimer’s and other dementias
ROC =: receiver operating curves
Simoa =: single molecule array
TDP-43 =: TAR DNA-binding protein 43
UMC =: University Medical Center; ZorgOnderzoek Nederland Medische Wetenschappen (ZonMw)
